# Role of Huangqin Decoction in Intestinal Homeostasis and Colon Carcinogenesis Based on “SREBP1 Cholesterol Metabolism Treg Cell Differentiation”

**DOI:** 10.1155/2023/6715978

**Published:** 2023-06-01

**Authors:** Junde Zhou, Nannan Lu, Xinxin Lv, Xin Wang, Jing Li, Lixia Ke

**Affiliations:** ^1^Ward 3 of General Surgery, The Second Affiliated Hospital of Harbin Medical University, No. 246 Xuefu Road, Nangang District, Harbin 150001, China; ^2^Department of Pathology, Bei'an First People's Hospital, No. 222, Longjiang Road, Bei'an 164099, China; ^3^Department of Oncology, Beidahuang Industry Group General Hospital, No. 235, Hashuang Road, Nangang District, Harbin 150001, China

## Abstract

**Objective:**

To explore the role of Huangqin Decoction in intestinal homeostasis maintenance and colon carcinogenesis based on “sterol regulatory element binding protein-1c (SREBP-1)-cholesterol metabolism regulatory T cell (Treg) differentiation.”

**Methods:**

It was decided to utilize a total of 50 healthy Wistar rats for the study, 20 of which were chosen at random to serve as controls, and 30 of which were used to create an intestinal homeostasis imbalance model. It was determined whether or not the modeling was successful by killing 10 rats from each of the two groups. The remaining 10 rats in the normal group were then employed as the control group for the experiment. The random number table method was used to split the rats into two groups: the Huangqin Decoction (*n* = 10) and the Natural Recovery (*n* = 10) groups. For seven days, participants in the Huangqin Decoction group received the herb, whereas those in the natural healing group received normal saline. The relative density of SREBP1, the levels of cholesterol ester (CE), free cholesterol (FC), total cholesterol (TC), and Treg cells were detected and compared.

**Results:**

When compared to the control group, the relative density of SREBP1 increased significantly before administration in the Huangqin Decoction group and the natural recovery group, but decreased significantly after administration, with statistical significance (*P* < 0.05) in the Huangqin Decoction group and the natural recovery group; the Huangqin Decoction group and natural recovery group had significantly higher levels of CE, FC, and TC than the control group before to administration, and these levels increased significantly after administration. CE, FC, and TC levels in Huangqin Decoction and natural recovery groups were much lower than those in natural recovery groups, and the difference was statistically significant (*P* < 0.05), according to the results; Prior to administration, Treg cell levels in Huangqin Decoction group and the natural recovery group were significantly higher, and Treg cell levels in the Huangqin Decoction group and natural recovery group were significantly lower after administration; the decrease in the Huangqin Decoction group was significantly greater than that in natural recovery group. *P* < 0.05 indicated that the difference was significant.

**Conclusion:**

Using Huangqin Decoction, one may efficiently regulate SREBP1, cholesterol metabolism, and Treg cell development, all of which play an important role in maintaining intestinal stability and minimizing the incidence of colon cancer.

## 1. Introduction

Intestinal homeostasis is a dynamic equilibrium state formed by the interaction of metabolites, nutrition, intestinal environment, and the host, which is affected by many factors such as eating habits, lifestyle, and environment [1]. As well as keeping the body's regular physiological functions in check, intestinal homeostasis is critical for preventing harmful microbe colonization, controlling immune system activity, and enhancing digestion and nutritional absorption [2]. Rheumatoid arthritis, central nervous system disorders, cardiovascular system diseases, endocrine system diseases, and digestive system diseases, including colon cancer, can all be caused by an imbalance [3]. Huangqin Decoction, from the Treatise on Febrile Diseases, has a variety of traditional Chinese medicine effects such as clearing away heat, neutralizing and relieving pain. It has a protective effect on the gastrointestinal tract and is commonly used to treat illnesses of the digestive system. In recent years, some scholars have pointed out that recent studies have found that Huangqin Decoction can not only reduce the death caused by chemotherapy in colon cancer model mice but also has preventive and therapeutic effects on colon cancer associated with colitis [4], and also plays an essential role in maintaining body homeostasis [5]. In addition, some studies have shown that sterol regulatory element-binding protein-1c (SREBP-1), cholesterol metabolism, and regulatory T cell (Treg) differentiation play an essential role in maintaining intestinal homeostasis [6–8]. However, few studies have explored the role of Huangqin Decoction in maintaining intestinal homeostasis and reducing colon cancer based on this aspect, which deserves further study. In order to better understand the role of Huangqin Decoction in maintaining intestinal homeostasis and preventing colon cancer, this study will investigate the “SREBP1-cholesterol metabolism-Treg cell differentiation” link between Huangqin Decoction and colon cancer.

## 2. Material and Methods

### 2.1. Selection and Grouping of Experimental Animals

50 clean-grade healthy Wistar rats (Beijing Vitonlihua Experimental Animal Co., Ltd., certificate number: SCXK 20060009) weighing 180 g to 220 g, 4 weeks old, and half male and half female, were selected [9].

### 2.2. Preparation of Huangqin Decoction

There are 12 significant dates in the Huangqin Decoction (1 big date weighs about 4 G) according to the proportion of Huangqin 9 g, Shaoyao 6 g, Liquorice 6 g, and it has a dosage of about 70 g [10]. The total amount of Huangqin Decoction is about 140 g, which is decocted according to the traditional Chinese medicine into a liquid medicine containing 1 g/ML of crude drug, and the residue is filtered with gauze and a 200 mesh nylon filter with a 0.22 *μ*m filter to remove bacteria in the clean table and kept at 4°C for later use.

### 2.3. Modeling, Grouping, and Administration

Lincomycin (Zhejiang Tianrui Pharmaceutical Co., Ltd., Standard Chinese Medicine H33022237) was administered to 20 Wistar rats from a total of 50 rats; the remaining 30 rats were given 0.3 g/mL lincomycin per day, a mouse model of intestinal homeostasis imbalance was established by 0.3 ml once and twice a day, in contrast, the control group received the same volume of sterile water [11]. To see if the modeling was successful, endotoxin, acetic acid, enterobacteriaceae, enterococcus, lactobacillus, and bifidobacterium were identified in the blood of 10 rats from each group after four days. Remaining normal-group rats were employed as controls, while the remaining 20 normal-group rats were divided into two groups: the Huangqin Decoction group (10 rats) and the natural recovery groups (ten rats). According to the random number table, the Huangqin Decoction group was given 0.3 ml Huangqin Decoction for gastric perfusion treatment, and the natural recovery group was given the same amount of saline for gastric perfusion treatment, 2 times a day for 7 days.

### 2.4. Detection Methods

#### 2.4.1. Detection of SREBP1 Expression by Western Blotting

Three groups of intestinal mucosal cells were collected before and 7 days after administration, and the cells were lysed with three decontamination lysates (Shanghai Kanglang Biotechnology Co., Ltd.), the precipitate was removed by centrifugation at 5000 r/min, and the protein content was measured using the BCA kit (Shanghai Yanxi Biotechnology Co., Ltd.), with the above sample buffer (Shanghai ABX Shanghai Biotechnology Co., Ltd.), the protein concentration of each group was adjusted to the same, and the gel was processed under 10% SDS-Polyacrylamide, after 2 hours of electrophoresis (120 V gel, 80 V gel), it was electrotransferred (100 MA, 4°C, 3 H) to the polyvinylidene fluoride (PDVF) membrane and dyed with Lichunhong (Hebei Pengyu Biotechnology Co., Ltd.), then the effect of the protein transfer was observed and the standard position of the protein molecular weight was determined. Rabbit anti-mouse SREBP1 was incubated at 37°C for 2 hours at a ratio of 1 : 1000 after being closed for 1 hour with 5% fat-free milk TBST (Shanghai Xuanke Biotechnology Co., Ltd.), then TBST was washed three times, and a 1 : 2,000 scale was added to the Horseradish peroxidase labeled sheep anti-rabbit II (Santa Cruz, USA). Following 0.5 hours of incubation at 37°C and three TBST washes, the samples were developed and fixed using a fluorescence detection kit (Shanghai Yanqi Biotechnology Co., Ltd.). The results were analyzed with an image analyzer (Shanghai Instrument & Electric Instrument Co., Ltd.).

#### 2.4.2. Cholesterol Test

Three sets of intestinal mucosal cells were collected before and after delivery; the cells were lysed with triple decontamination lysate, and the precipitates were removed by centrifugation at 5000 r/min. The buffer solution was used to get the protein concentration in each group down to 1.0 g/L, and then the medium was discarded. The lytic cells were frozen and thawed 3 times using PBS buffer solution (Semmerphisil Technology Co., Ltd.) and 500 *μ*L 0.1 mol/l sodium hydroxide solution (Zhengzhou Yongkun Environmental Protection Technology Co., Ltd.). After quantifying the protein content with the BCA reagent, the protein was precipitated with 7.2% trichloroacetic acid. The supernatant was centrifuged at 800 × g for 10 min. Taking stigmasterol as the internal standard and making the standard curve, the supernatant of 100 *μ*L was added to 200 *μ*L 8.9 mol/L potassium hydroxide, total cholesterol (TC) was obtained by hydrolysis of cholesteryl ester (CE), and free cholesterol (FC) was obtained by adding 200 *μ*L 1 mol/l sodium hydroxide. The samples were mixed separately with the internal standard solution and then extracted with anhydrous ethanol (Yucheng haowei Chemical Co., Ltd.) and N-hexane (Shandong Weiming Chemical Co., Ltd.), 1.5 chromium trioxide, and vacuum drying. The samples were dissolved by 80 *μ*L Yiqing (Shandong Hongcheng Chemical Co., Ltd.) and 20 *μ*L isopropanol (Wuxi Dongneng Chemical Technology Co., Ltd.) through the high-performance liquid chromatography (Shanghai Wufeng Scientific Instruments Co., Ltd.) on the sample. C18 column (Shanghai Weixi Biological Technology Co., Ltd.) was used for the detection. UV light at 250 nm, flow rate of 1 ml/min, column temperature of 4°C, TC was quantified by peak area and calibrated by an internal standard, mg/G cell protein. CE = TC-FC.

#### 2.4.3. Treg Cell Test

After drawing 2 mL of anticoagulant blood from each group, the erythrocytic-lysate solution (Hangzhou Haoxin Biotechnology Co., Ltd.) was added, and the mixture was uniformly mixed before being incubated for 15 minutes at room temperature, the rat anti-human FITC-CD4 antibody (Shanghai Bangjing Industrial Co., Ltd.) was added with 1 × 105 cells/L after washing twice with PBS buffer. The cells were then rinsed with PBS buffer and treated for 30 minutes at 4°C with rabbit anti-human PE-CD25 antibody (Shanghai Wanjiang Biotechnology Co., Ltd.) and rat anti-human Foxp3-PE-Cy5 antibody (Beijing Boao Pike Biotechnology Co., Ltd.), followed by Flow cytometry cells and CD4CD25FOXP3 + cells. Finally, cell quest V 3.2 the proportion of Treg cells in the total CD4 + T lymphocyte was analyzed.

### 2.5. Observational Index

① Compare the relative density of SREBP1 before and after administration in each group; ② Compare CE, FC and TC levels in each group before and after administration; and ③ Compare Treg cell levels in each group before and after administration.

### 2.6. Statistical Methods

In the statistical study, SPSS 18.0 was utilized, and the mean ± standard deviation (±*s*) was employed to represent the data, *T* was used to test, *N* or% was used to express the data, Χ2 was used to test. Using the *F* value, the difference between the two groups was tested, and *P* < 0.05 was utilized to signify significance.

## 3. Outcome

### 3.1. Comparison of SREBP1 Relative Density before and after Administration in Each Group

**Figure 1:**
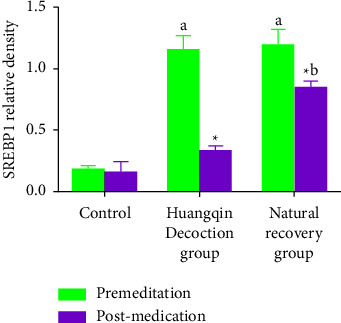
Comparison of SREBP1 relative density before and after administration in each group.

**Figure 2:**
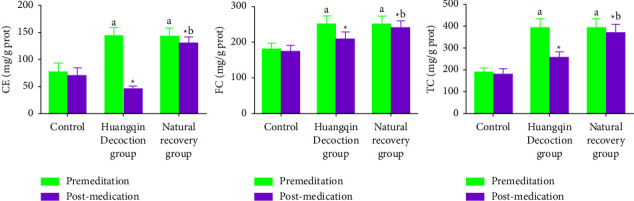
Comparison of CE, FC, and TC levels before and after administration in each group.

**Figure 3:**
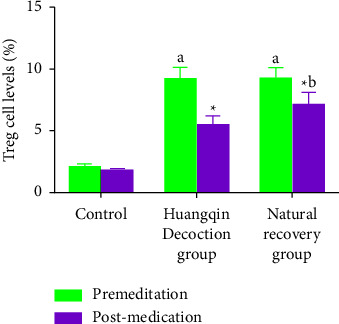
Comparison of Treg cell levels before and after administration in each group.

There was a significant difference (*P* < 0.05) in the relative density of SREBP1 between the Huangqin Decoction and natural recovery groups and the control group before delivery, and after administration, SREBP1 concentrations were considerably lower in the Huangqin Decoction group than in the natural recovery group.  Table 1 shows that the Huangqin Decoction group's decline range was considerably greater than that of the natural recovery group (*P* < 0.05) Figures 1, 2, 3, 13.

### 3.2. Comparison of CE, FC, and TC Levels before and after Administration in Each Group

Before administration, the levels of CE, FC, and TC in Huangqin Decoction group and natural recovery group were significantly higher than those in control group (*P* < 0.05), and the levels of CE, FC, and TC in Huangqin Decoction group and natural recovery group were significantly lower after administration, the decrease range of Huangqin Decoction group was significantly greater than that of natural recovery group (*P* < 0.05), see Table 2.

### 3.3. Comparison of Treg Cell Levels before and after Administration in Each Group

In the Huangqin Decoction and natural recovery groups, the Treg cell level was considerably greater before administration (*P* < 0.05) than that in the control group, and after administration, the Treg cell level was significantly reduced in the Huangqin Decoction and natural recovery groups, According to Table 3, there was a significant difference between the Huangqin Decoction and the natural recovery groups (*P* < 0.05).

## 4. Discussion

The role of intestinal homeostasis in the vital activities of the body has received increasing attention in clinical practice; there are significant differences in the composition of intestinal microflora between colorectal cancer patients and noncolorectal cancer patients, implying that homeostasis is closely related to the occurrence of colon cancer. Because of the high density of intestinal flora in the colon, when it is out of balance, it can cause the incidence and development of colon disease. Intestinal homeostasis imbalance can result in intestinal mucosal immune function imbalance, the decrease of intestinal short-chain fatty acid, the metabolic disorder of intestinal bile acid, the increase of susceptibility to carcinogens and the proliferation of colonic epithelium. In addition, bile acids can produce carcinogenic metabolites under the action of intestinal microflora, which promote the development of colon cancer. Huangqin Decoction group rigorous, with Huangqin qingre detoxification, peony astringent and ying pain, adjuvant medicine licorice, so that the Chinese date and in the first, four drugs combined, play Qingre dysentery, slow pain relief effect. Pharmacological studies show that Huangqin Decoction has obvious antibacterial, anti-inflammatory, spasmolytic, analgesic, antipyretic, enhancing immunity, sedative and other effects. It has essential implications for maintaining intestinal homeostasis [12]. Aside from this, the effect of Huangqin Decoction in the preservation of intestinal homeostasis and the development of colon cancer on the basis of “SREBP1-cholesterol metabolism-Treg cell differentiation” has not yet been investigated.

SREBP1 is composed of DNA binding subunits and regulatory subunits, located in the endoplasmic reticulum, which is an essential regulatory protein for cholesterol metabolism and membrane binding transcription factors in cells [13]. Abnormal blood lipids can cause upregulation of SREBP1, however, Tongmai Jiangzhuo decoction (containing Bupleurum Root 15 g, radix scutellariae 9 g, radix et rhizoma Rhei 6 g, radix paeoniae alba 9 g, jujube 9 g, Pinellia ternata 9 g) could effectively downregulate the expression of SREBP1, before administration, the relative density of SREBP1 in Huangqin Decoction group and natural recovery group was higher than that in the control group [14, 15]. After administration, the relative density of SREBP1 in the Huangqin Decoction group was lower than that in the control group and the SREBP1 was upregulated in the group of Huangqin Decoction and upregulated in the group of Huangqintang, which is significant for maintaining intestinal homeostasis and reducing the risk of colon cancer. Metabolic abnormality is an essential feature of intestinal homeostasis imbalance and tumor cells, which is characterized by hyperactive cholesterol oxidation, alteration of related metabolic enzymes, and enhancement of tumor signaling pathways [16]. SREBP1, a key transcription factor regulating lipid metabolism, regulates gene expression of key enzymes in fatty acid and cholesterol synthesis and plays a regulatory role in lipid regeneration. The expression and activity of SREBP1 in normal cells and tissues are low. SREBP1 can be activated in hard egg tumors and promote the expression of genes downstream of SREBP1. SREBP1 can not only accelerate the synthesis of cholesterol and fatty acids; moreover, it can also affect the metabolism of amino acid, glucose and lipid of tumor cells through various signal pathways, providing certain material and energy for tumor cell metastasis and proliferation [17]. Baicalin, Paeoniflorin, and other effective components in Huangqin Decoction can not only effectively interfere with the immune response of the body but also regulate the intestinal microorganisms [18]. It has been suggested that Huangqin Decoction can increase tumor cell necrosis, promote monocyte to M1 transformation and regulate microenvironment; as a result, preventing colon cancer by maintaining intestinal homeostasis is extremely important.

Studies by Moriya et al. [19] show that the TC level of colorectal cancer is significantly increased after the occurrence of colorectal cancer, which is closely related to the tumor stage, the ability of proliferation, and the degree of invasion. Both the Huangqin Decoction and natural recovery groups had significantly increased levels of CE, FC, and TC compared to the control group. After administration, the levels of CE, FC, and TC in the two groups decreased. Huangqin Decoction, on the other hand, decreased more, which was in line with the results of Ding Xiaorui and izhenwei, who found that it had a similar effect, indicating that the imbalance of intestinal homeostasis could cause abnormal metabolism of cholesterol and that Scutellaria baicalensis can correct its metabolic disorder. As tumor invasion progresses, cholesterol's role in cancer formation and progression becomes increasingly important., the higher the stage of tumor and the stronger the ability of cell division and proliferation, the more cholesterol is required. In order to maintain intestinal homeostasis and prevent the incidence of colon cancer, the glycosides in Huangqin Decoction have favorable effects on decreasing blood lipid and blood pressure.

The results of Moriya et al. [19] showed that the level of Treg in colon cancer patients with intestinal obstruction increased, but after effective treatment, the level of Treg decreased effectively. Fan et al. [20] showed that Huangqin Decoction could regulate the level of Treg in the body and regulate the homeostasis of intestinal immunity, treg cell level decreased in the two groups after administration, but the decrease was more obvious in the Huangqin Decoction group than in the natural recovery group, which was consistent with the results of Du et al. and Zhao et al. the Huangqin Decoction group could effectively regulate the differentiation of Treg cells [21, 22]. Treg, as an immune cell, can inhibit the differentiation of immunocompetent cells by direct action between cells. The activity of numerous inflammatory substances can also hinder the immune response against tumors, baicalein, Baicalin, and Paeonia lactiflora pall in the Huangqin Decoction can effectively regulate the immune system, therefore, anti-inflammatory properties of Huangqin Decoction help preserve intestinal homeostasis and minimize colon cancer risk [23, 24].

To summarize, Huangqin Decoction has a high clinical reference value for maintaining intestinal homeostasis and reducing colon cancer risk through regulation of SREBP1, cholesterol metabolism, and Treg cell development in the body.

## Figures and Tables

**Table 1:** Comparison of SREBP1 relative density before and after administration in each group (±*s*).

Time	Control group (*n* = 10)	Huangqin Decoction group (*n* = 10)	Natural recovery group (*n* = 10)	*F*/*t* value	*P* value
Premeditation	0.18 ± 0.03	1.15 ± 0.12	1.19 ± 0.13	6.524	0.017
Postmedication	0.15 ± 0.09	0.33 ± 0.04^*∗*^	0.84 ± 0.06^*∗*^	5.473	0.032

Note: ^*∗*^indicates the comparison between the same groups before and after administration, ^*∗*^*P* < 0.05.

**Table 2:** Comparison of CE, FC, and TC levels before and after administration in each group (mg/g prot, ±*s*).

Measurements		Control group (*n* = 10)	Huangqin Decoction group (*n* = 10)	Natural recovery group (*n* = 10)	*F*/*t* value	*P* value
CE	Premedication	77.02 ± 16.26	143.40 ± 15.01	142.68 ± 15.27	18.026	0.000
	Postmedication	69.98 ± 14.96	45.28 ± 5.50^*∗*^	130.23 ± 11.23^*∗*^	7.633	0.002

FC	Premedication	181.32 ± 16.20	250.91 ± 23.16	251.10 ± 23.15	8.431	0.000
	Postmedication	173.62 ± 18.93	210.21 ± 19.52^*∗*^	240.17 ± 21.31^*∗*^	7.018	0.010

TC	Premedication	188.23 ± 21.61	393.16 ± 40.25	393.31 ± 40.13	9.142	0.000
	Postmedication	180.36 ± 23.69	255.84 ± 26.02^*∗*^	370.33 ± 38.98^*∗*^	7.809	0.000

Note: ^*∗*^indicates the comparison between the same groups before and after administration, ^*∗*^*P* < 0.05.

**Table 3:** Comparison of Treg cell levels before and after administration in each group (%, ±*s*).

Time	Control group (*n* = 10)	Huangqin Decoction group (*n* = 10)	Natural recovery group (*n* = 10)	*F*/*t* value	*P* value
Premeditation	2.09 ± 0.27	9.15 ± 1.00	9.21 ± 0.92	6.518	0.020
Postmedication	1.78 ± 0.16	5.45 ± 0.75^*∗*^	7.11 ± 1.00^*∗*^	5.162	0.039

Note: ^*∗*^indicates the comparison between the same groups before and after administration, ^*∗*^*P* < 0.05;

## Data Availability

The data used to support the findings of this study are available from the corresponding author upon request.
